# Draft Genome Sequences of a Bacillus subtilis Strain, a Bacillus velezensis Strain, a *Paenibacillus* Strain, and an Acinetobacter baumannii Strain, All Isolated from the Phyllosphere of Lactuca sativa or Solanum lycopersicum

**DOI:** 10.1128/MRA.01092-20

**Published:** 2021-01-28

**Authors:** Claudia Y. Muñoz, Anne de Jong, Oscar P. Kuipers

**Affiliations:** a Department of Molecular Genetics, University of Groningen, Groningen, the Netherlands; University of Arizona

## Abstract

Four strains isolated from tomato and lettuce phyllosphere were sequenced in order to investigate the presence of novel antimicrobial gene clusters and to get a better understanding of plant microbe interactions. These strains comprise two *Bacillus* strains, one *Paenibacillus* strain, and one *Acinetobacter* strain.

## ANNOUNCEMENT

Crop production is facing several challenges, such as a higher demand due to an increased population, climate change, and yield loss caused by pathogens. The extensive use of chemical pesticides in agricultural production has caused negative effects including contamination of soils and water and toxicity in animals, including humans (1). Beneficial microbes in the plant microbiome have shown good potential for application as pathogen control and plant growth promoters in order to increase crop yields. Therefore, we can regard them as environmentally friendly alternatives to chemical products in agricultural practices (2).

*Bacillus* species are Gram-positive, spore-forming bacteria widely distributed in the environment; they are well known as producers of a wide array of antimicrobials, having between 5 and 8% of the total genome devoted to the biosynthesis of secondary metabolites. (3). *Paenibacillus* is another Gram-positive bacterium that has shown major positive effects in sustainable agriculture, medicine, and biotechnology (4); thus, *Bacillus* and *Paenibacillus* have become attractive biological control agents. Finally, during the last 2 decades, Gram-negative *Acinetobacter* bacteria have been reported for the first time as new possible biocontrol agents. However, some species, such as Acinetobacter baumannii, are highly pathogenic for humans (5).

Four possible biocontrol strains were isolated from the phyllosphere of healthy tomato and lettuce crops in Groningen, the Netherlands. Briefly, 2 g of leaves from tomato and lettuce were macerated to a homogenous liquid state using 5 ml of 10 mM MgSO_4_ buffer. Serial dilutions were prepared, and 1 ml of each dilution was exposed to heat treatment of 80°C for 15 min. After the heat treatment, the dilutions were spread onto separate LB agar plates and incubated at 28°C for the next 72 h. Colony growth was monitored every day, and each colony was isolated and cultured on a separate plate. The isolated colonies were finally grown overnight in liquid LB medium at 28°C with shaking at 220 rpm, and stocks were created by using glycerol at 80% solutions with 720 μl of culture and 750 μl of glycerol and stored at −80°C.

For genome sequencing, strains from the −80°C glycerol stocks were streaked onto LB agar plates, and a single colony of each strain was grown in 3 ml of LB medium at 28°C with shaking at 220 rpm. Overnight cultures of the 4 strains in LB medium were collected by centrifugation.

Genomic DNA was isolated with a GenElute bacterial genomic DNA isolation kit (Sigma-Aldrich) according to the manufacturer’s protocol. The genomes were sequenced at BGI Tech Solutions (Hong Kong), with an Illumina HiSeq sequencing system. On average, 5 million paired-end raw reads (150 bp) were generated per sample. Fast QC version 0.11.9 (6) was used to examine the quality of the reads, and low-quality reads were removed with Trimmomatic version 0.38 (7). The reads were assembled *de novo* using SPAdes version 3.11.1 with default parameters (8). The genome sequences were annotated by the NCBI using the Prokaryotic Genome Annotation Pipeline (PGAP) and are publicly available (9).

Within the manuscript, the draft genome sequences were annotated with the Rapid Annotations using Subsystems Technology (RAST) server and identified as *Bacillus*, *Paenibacillus*, and Acinetobacter by phylogenetic analysis with available whole-genome sequences (10). Genome mining was conducted with antiSMASH, showing potential novel nonribosomal peptides (NRPs) and polyketides (PKs), which are under investigation (11).

### Data availability.

The draft genome sequences of the 4 strains have been deposited in GenBank under the accession numbers listed in Table 1. The raw reads have been registered and submitted to the Sequence Read Archive (SRA) under the accession numbers listed in Table 1.

**TABLE 1:** Genome features and GenBank accession numbers of the strains

Strain	Genome size (bp)	G+C content (%)	No. of coding sequences	*N*_50_ (bp)	No. of contigs	Coverage (×)	No. of RNAs	GenBank accession no.	SRA accession no.
Bacillus subtilis STRP31	4,362,346	43.9	4,987	1,049,484	931	>150	97	JABBYF000000000	SRX9245527
Bacillus velezensis SPL51	4,390,708	45.5	4,522	229,106	57	>150	70	JABBYI000000000	SRX9245528
*Paenibacillus* sp. strain PL91	8,014,288	47.3	7,583	409,462	80	>150	65	JABBYG000000000	SRX9234476
Acinetobacter baumannii PL81	4,001,457	38.9	3,575	221,958	40	>150	66	JABBYH000000000	SRX9234475

## References

[1] Compant S, Samad A, Faist H, Sessitsch A 2019 A review on the plant microbiome: ecology, functions, and emerging trends in microbial application. J Adv Res 19:29–37. doi:10.1016/j.jare.2019.03.004. 31341667 PMC6630030

[2] Levy A, Conway JM, Dangl JL, Woyke T 2018 Elucidating bacterial gene functions in the plant microbiome. Cell Host Microbe 24:475–485. doi:10.1016/j.chom.2018.09.005. 30308154

[3] Fira D, Dimkić I, Berić T, Lozo J, Stanković S 2018 Biological control of plant pathogens by Bacillus species. J Biotechnol 285:44–55. doi:10.1016/j.jbiotec.2018.07.044. 30172784

[4] Grady EN, MacDonald J, Liu L, Richman A, Yuan Z-C 2016 Current knowledge and perspectives of *Paenibacillus*: a review. Microb Cell Fact 15:203. doi:10.1186/s12934-016-0603-7. 27905924 PMC5134293

[5] Xue Q-Y, Chen Y, Li S-M, Chen L-F, Ding G-C, Guo D-W, Guo J-H 2009 Evaluation of the strains of Acinetobacter and Enterobacter as potential biocontrol agents against Ralstonia wilt of tomato. Biol Control 48:252–258. doi:10.1016/j.biocontrol.2008.11.004.

[6] Andrews S 2010 FastQC: a quality control tool for high throughput sequence data. http://www.bioinformatics.babraham.ac.uk/projects/fastqc/.

[7] Bolger AM, Lohse M, Usadel B 2014 Trimmomatic: a flexible trimmer for Illumina sequence data. Bioinformatics 30:2114–2120. doi:10.1093/bioinformatics/btu170. 24695404 PMC4103590

[8] Bankevich A, Nurk S, Antipov D, Gurevich AA, Dvorkin M, Kulikov AS, Lesin VM, Nikolenko SI, Pham S, Prjibelski AD, Pyshkin AV, Sirotkin AV, Vyahhi N, Tesler G, Alekseyev MA, Pevzner PA 2012 SPAdes: a new genome assembly algorithm and its applications to single-cell sequencing. J Comput Biol 19:455–477. doi:10.1089/cmb.2012.0021. 22506599 PMC3342519

[9] Tatusova T, DiCuccio M, Badretdin A, Chetvernin V, Nawrocki EP, Zaslavsky L, Lomsadze A, Pruitt KD, Borodovsky M, Ostell J 2016 NCBI Prokaryotic Genome Annotation Pipeline. Nucleic Acids Res 44:6614–6624. doi:10.1093/nar/gkw569. 27342282 PMC5001611

[10] Aziz RK, Bartels D, Best AA, DeJongh M, Disz T, Edwards RA, Formsma K, Gerdes S, Glass EM, Kubal M, Meyer F, Olsen GJ, Olson R, Osterman AL, Overbeek RA, McNeil LK, Paarmann D, Paczian T, Parrello B, Pusch GD, Reich C, Stevens R, Vassieva O, Vonstein V, Wilke A, Zagnitko O 2008 The RAST server: Rapid Annotations using Subsystems Technology. BMC Genomics 9:75. doi:10.1186/1471-2164-9-75. 18261238 PMC2265698

[11] Blin K, Wolf T, Chevrette MG, Lu X, Schwalen CJ, Kautsar SA, Suarez Duran HG, de Los Santos ELC, Kim HU, Nave M, Dickschat JS, Mitchell DA, Shelest E, Breitling R, Takano E, Lee SY, Weber T, Medema MH 2017 antiSMASH 4.0: improvements in chemistry prediction and gene cluster boundary identification. Nucleic Acids Res 45:W36–W41. doi:10.1093/nar/gkx319. 28460038 PMC5570095

